# Endoscopic findings in patients with Shwachman–Diamond syndrome: A report from the North American Shwachman–Diamond syndrome registry

**DOI:** 10.1002/jpr3.70218

**Published:** 2026-07-05

**Authors:** Elizabeth Korn, Jane Koo, Diana Schwarz, Meghan Haney, Juan Putra, Jaeson Kim, Leah Cheng, Sarah Steltz, Lois Schwarz, Sara Loveless, Richard Cooper, Claire Dusa, Kasiani C. Myers, Akiko Shimamura, Andrew Wehrman, Amit S. Grover

**Affiliations:** ^1^ Department of Pediatrics, Harvard Medical School Division of Pediatric Hematology/Oncology, Dana‐Farber/Boston Children's Cancer and Blood Disorders Center Boston Massachusetts USA; ^2^ Department of Pediatrics, Division of Bone Marrow Transplantation and Immune Deficiency, Cincinnati Children's Hospital Medical Center University of Cincinnati Cincinnati Ohio USA; ^3^ Department of Pathology Boston Children's Hospital, and Harvard Medical School Boston Massachusetts USA; ^4^ Division of Gastroenterology, Hepatology and Nutrition, Boston Children's Hospital Boston Massachusetts USA; ^5^ Clinical Research Center, Boston Children's Hospital Boston Massachusetts USA

**Keywords:** bone marrow failure, colonoscopy, endoscopy, gastrointestinal

## Abstract

**Objectives:**

Shwachman–Diamond syndrome (SDS) is an inherited bone marrow failure disorder, and its endoscopic phenotype is poorly defined. We sought to characterize endoscopic findings in patients with genetically confirmed SDS.

**Methods:**

Retrospective registry study of 45 patients with biallelic Shwachman–Bodian–Diamond syndrome mutations and evaluable endoscopic procedures. Clinical, laboratory, imaging, endoscopic reports, and biopsy findings were extracted from patient medical records.

**Results:**

Among 45 patients with genetically confirmed SDS, 102 endoscopic procedures were performed, most commonly esophagogastroduodenoscopy (EGD, 58.8%). The leading indication was diarrhea and/or steatorrhea (23.6%). Abnormal biopsy findings were most frequent in patients with dysphagia/dyspepsia or gastrointestinal bleeding and were strongly associated with elevated C‐reactive protein and abnormal hepatic enzymes. Most pre‐procedural imaging studies were normal (63.4%). Pre‐hematopoietic stem cell transplant (HSCT) biopsies were largely normal, with occasional mild acute or chronic inflammation in the esophagus, stomach, and duodenum. In contrast, post‐HSCT biopsies showed more diverse abnormalities, including inflammation, basal crypt apoptosis, and graft‐versus‐host disease (GVHD)–associated changes. Central pathology review also identified rare findings such as adenoviral infection and mild or equivocal GVHD in post‐transplant samples.

**Conclusions:**

Endoscopic evaluation in patients with SDS demonstrates heterogeneous histopathologic findings, with diagnostic yield influenced by clinical indication, laboratory abnormalities, and transplant status. Routine endoscopy in asymptomatic or nonspecifically symptomatic patients may have limited value, whereas targeted evaluation based on symptoms or supportive laboratory findings may improve diagnostic yield. These findings provide updated insight into gastrointestinal manifestations of SDS and may help guide clinical decision‐making regarding endoscopic evaluation in this rare disease.

## Abstract (highlights)

**What is Known:**

- Shwachman–Diamond syndrome (SDS) is a rare inherited bone marrow failure disorder classically associated with exocrine pancreatic dysfunction.
- Gastrointestinal manifestations in SDS primarily reflect exocrine pancreatic dysfunction, though rare enteropathies such as celiac disease or inflammatory bowel disease have been reported; contemporary endoscopic data remain extremely limited.

**What is New:**

- In the largest genetically confirmed SDS cohort with systematically evaluated endoscopic findings, diagnostic yield varied by presenting symptom, laboratory markers, and hematopoietic stem cell transplant (HSCT) status—suggesting routine endoscopy in asymptomatic patients have limited utility.
- Elevated fecal calprotectin and liver enzymes were associated with higher rates of abnormal histopathology, supporting their role in risk‐stratifying patients for endoscopic evaluation.
- Post‐HSCT biopsies demonstrated more frequent and heterogenous abnormalities—including changes due to graft‐vs‐host‐disease—highlighting the importance of distinguishing transplant‐related gastrointestinal pathology from primary SDS‐associated disease.

## 1 INTRODUCTION

Shwachman–Diamond syndrome (SDS) is an inherited bone marrow failure syndrome classically characterized by neutropenia, exocrine pancreatic dysfunction (PED), and a predisposition toward progressive bone marrow failure and elevated risk of hematologic malignancies.^1^, ^2^ SDS is a disorder of ribosomal biogenesis and approximately 90% of patients with SDS have biallelic pathogenic variants in the Shwachman–Bodian–Diamond syndrome (*SBDS*) gene located on chromosome 7q11.^3^ Hematopoietic stem cell transplantation (HSCT) remains the only curative therapy option for patients with SDS who develop bone marrow failure or transform to myeloid malignancy.^4^

Gastrointestinal manifestations classically reflect PED with diarrhea or steatorrhea, though rare enteropathies resembling celiac disease^5^ or inflammatory bowel disease^6^ have been reported. Contemporary data on endoscopic findings in SDS are extremely limited.^7^ We therefore sought to systematically characterize endoscopic findings in patients with SDS enrolled in the North American Shwachman–Diamond syndrome Registry (SDSR).

## 2 METHODS

### 2.1 Ethics statement

Informed consent was obtained from all participants or their legal guardians. Approval from the Cincinnati Children's Hospital Medical Center and Boston Children's Hospital Institutional Review Boards approval was obtained at both participating institutions (Approved March 8, 2025).

### 2.2 Patient cohort

We conducted a retrospective registry‐based cohort study using medical records of patients with biallelic *SBDS* mutations enrolled in the North American SDS Registry between January 2009 and December 2024 with informed consent.

### 2.3 Laboratory, radiographic, and endoscopic data

We reviewed all available endoscopic‐related clinical, laboratory, radiographic, and histopathologic data. For laboratory parameters, normal reference values were defined as fecal calprotectin <50 µg/g, C‐reactive protein (CRP) <2 mg/L, and liver enzymes within age‐ and sex‐specific normative ranges.^8^, ^9^ Procedure‐level data (e.g., indications, gross/endoscopic findings) were extracted from endoscopy procedure reports. Histologic findings were extracted from pathology reports when biopsies were obtained and corresponding reports were available within the registry; as such, the number of procedures with histologic data is lower than the total number of procedures performed. HSCT itself is a known risk factor for gastrointestinal and hepatic complications, and findings observed after transplantation may therefore reflect transplant‐related sequelae rather than the underlying manifestations of SDS. Therefore, histopathologic results from endoscopic procedures were stratified by transplantation status.

### 2.4 Statistical analysis

Descriptive statistics were used to summarize patient characteristics, indications for endoscopy, laboratory findings, imaging results, and histopathologic outcomes. Categorical variables were reported as frequencies and percentages. Endoscopic and biopsy findings were stratified by gastrointestinal segment and transplant status (pre‐HSCT vs. post‐HSCT). The frequency of abnormal biopsy findings was evaluated according to clinical indication and relevant laboratory abnormalities. Analyses were primarily descriptive given the exploratory nature of the study and the small sample size. Analyses were conducted in GraphPad Prism 10.5.0.

## 3 RESULTS

Patient demographics of the 45 patients with genetically confirmed biallelic *SBDS* mutations are described in Table 1. Median age of diagnosis of SDS was 2.4 years old (range 0.3–38.7 years). Twenty‐one (46.7%) required HSCT. Twenty‐one (46.7%) required HSCT, of whom nine (42.9%) underwent endoscopic evaluation prior to transplant, all performed for gastrointestinal symptoms. In our cohort the most common presenting gastrointestinal symptoms prior to diagnosis of SDS was failure to thrive (FTT, *n* = 30, 41.7%) followed by diarrhea/steatorrhea (*n* = 19, 26.4%).

**Table 1:** Patient demographics and clinical characteristics.

	Patients (*n* = 45)
Sex, *n* (%)	
Female	31 (68.9)
Male	14 (31.1)
Race, *n* (%)	
White	39 (86.7)
Asian	1 (2.2)
Mixed	1 (2.2)
Unknown	4 (8.9)
*SBDS* mutations, *n* (%)	
c.258+2T>C; c.183_184TA>CT	25 (55.6)
c.258+2T>C; c.258+2T>C	7 (15.6)
c.258+2T>C; c.250T>C	2 (4.4)
c.258+2T>C; c.183_184TA>CT and c.258+2T>C	1 (2.2)
c.258+2T>C; c.120delG	1 (2.2)
c.258+2T>C; c.184A>T	1 (2.2)
c.258+2T>C; c.258+1G>C	1 (2.2)
c.258+2T>C; c.259‐2A>G	1 (2.2)
c.258+2T>C; c.460‐1G>A	1 (2.2)
c.258+2T>C; c.523delC	1 (2.2)
c.258+2T>C; c.641C>T	1 (2.2)
c.258+2T>C; c.664G>C	1 (2.2)
c.258+2T>C; c.171dup	1 (2.2)
c.258+2T>C; 616_619del	1 (2.2)
Median age at diagnosis of SDS in years, (range)	2.4 (0.3–38.7)
HSCT, *n* (%)	21 (46.7)
Median age at HSCT in years, (range)	16.4 (0.5–39.7)
Presenting gastrointestinal symptoms prior to SDS diagnosis, *n* (%)	*n *= 45*
Abdominal pain	4 (8.9)
Diarrhea/steatorrhea	19 (42.2)
Elevated liver enzymes	7 (15.6)
Pancreatic dysfunction	12 (26.7)
Failure to thrive	30 (66.7)
Survival, *n* (%)	
Alive at last follow‐up	39 (86.7)

Abbreviations: HSCT, hematopoietic stem cell transplant; SBDS: Shwachman–Bodian–Diamond syndrome; SDS, Shwachman–Diamond syndrome.

*Number of patients with presenting symptoms.

Endoscopic procedures are frequently performed in the post‐HSCT period to assess for complications such as graft‐versus‐host disease (GVHD) or infection; therefore, procedures were categorized as pre‐HSCT (including untransplanted patients) or post‐HSCT (Table 2). A total of 102 endoscopic procedures were performed, of which the majority were esophagogastroduodenoscopies (EGD, *n* = 60, 58.8%), 21 (20.6%) colonoscopies and 18 (17.6%) sigmoidoscopies. Small bowel endoscopy (*n* = 2) and wireless capsule endoscopy comprised (*n* = 1) only 3% of all procedures. Median age at time of endoscopy was 10 years old (range 0.3–40.4 years). Median time of endoscopic procedure from confirmed SDS diagnosis was 1.7 years (range −3.6 years prior to diagnosis to 20 years after diagnosis). Fourteen (13.7%) procedures were performed prior to SDS diagnosis for work‐up of abdominal pain, diarrhea/steatorrhea, reflux, and FTT. No complications were reported intra‐operatively in procedures (*n* = 54, 100%).

**Table 2:** Characteristics of endoscopies in patients with Shwachman–Diamond syndrome.

	All procedures (*n* = 102)	Pre‐HSCT/No HSCT (*n* = 69)	Post‐HSCT (*n* = 33)
Procedure, *n* (%)			
EGD	60 (58.8)	48 (69.6)	12 (36.4)
Colonoscopy	21 (20.6)	11 (15.9)	10 (30.3)
Sigmoidoscopy	18 (17.6)	10 (14.5)	8 (24.2)
Small bowel enteroscopy	2 (2)	0	2 (6.1)
WCE	1 (1)	0	1 (3)
Indication for endoscopy, *n* (%)^a^	*n *= 89^c^	*n *= 54	*n *= 35
Diarrhea/steatorrhea	21 (23.6)	11 (20.4)	10 (28.6)
Abdominal pain	14 (15.7)	12 (22.2)	2 (5.7)
Failure to thrive/weight loss	14 (15.7)	12 (22.2)	2 (5.7)
Nausea/vomiting	10 (11.2)	6 (11.1)	4 (11.4)
Dysphagia	5 (5.6)	4 (7.4)	1 (2.9)
Reflux	5 (5.6)	3 (5.6)	2 (5.7)
Globus/chest pain	3 (3.4)	2 (3.7)	1 (2.9)
Anemia	3 (3.4)	1 (1.9)	2 (5.7)
Gastrointestinal bleeding	2 (2.2)	2 (3.7)	0
Mass/surveillance of polyp	2 (2.2)	1 (1.9)	1 (2.9)
Evaluation for GVHD	4 (4.4)	0	4 (11.4)
Other^b^	4 (4.4)	0	0
Unknown	2 (2.2)	0	2 (5.7)
Median age of endoscopy in years (range)	10.0 (0.3–40.4)	7.0 (0.3–40.4)	10.9 (0.6–32.2)
Median time in years of endoscopy from SDS diagnosis (range)	1.7 (−3.6 to 20.7)	0.03 (−3.6 to 20.7)	6.7 (0.3–16.6)
Medications administered during endoscopy, *n* (%)	*n* = 83	*n* = 41	*n* = 42
Pancreatic enzyme replacement	38 (45.8)	20 (48.8)	18 (42.9)
Proton pump inhibitor/H2 blocker	27 (32.5)	20 (48.8)	7 (16.7)
Steroid	7 (8.4)	1 (2.4)	6 (14.3)
Immune suppression^d^	10 (12)	0	10 (23.8)
Mucosal protective agent	1 (1.2)	0	1 (2.4)
Laboratory results prior to endoscopy, *n* (%)			
Elevated CRP (*n *= 22^!^)	6 (27.3)	*n* = 16^!^, 4 (25)	*n* = 6^!^, 2 (33.3)
Elevated fecal calprotectin (*n* = 7^!^)	5 (71.4)	*n* = 3^!^, 1 (33.3)	*n* = 4^!^, 4 (100)
Elevated liver enzymes (*n* = 54^!^)	20 (37)	*n* = 17^!^, (88.2)	*n* = 22^!^, 5 (22.7)
Positive hemoccult (*n* = 4^!^)	1 (25)	*n* = 2^!^, (0)	*n* = 2^!^, 1 (50)
Positive infection (*n* = 9^!^)	1 (11.1)	*n* = 5^!^, (0)	*n* = 4^!^, 1 (25)
Imaging obtained prior to endoscopy, *n* (%)	*n* = 41 patients	*n* = 24	*n* = 17
Ultrasound	17 (41.5)	14 (58.3)	3 (17.6)
CT	10 (24.4)	6 (25)	4 (23.5)
MRI	5 (12.2)	1 (4.2)	4 (23.5)
X‐ray/upper GI series	9 (21.9)	3 (12.5)	6 (35.2)
Proportion of normal imaging by modality, *n* (%)			
Ultrasound	6 (35.3)		
CT	6 (60)	*‐*	*‐*
MRI	4 (100)		
X‐ray/upper GI series	9 (100)		
Imaging results prior to endoscopy, *n* (%)	*n* = 41	*n* = 24	*n* = 17
Normal	26 (63.4)	11 (45.8)	15 (88.2)
Pancreatic echogenicity/pancreatic fatty replacement	10 (24.4)	9 (37.5)	1 (5.9)
Liver echotexture changes	2 (4.9)	1 (4.2)	1 (5.9)
Other^e^	3 (7.3)	3 (12.5)	0
Intra‐operative complications, *n* (%)	*n = 54*		
None	54 (100)	33 (100)	21 (100)21

Abbreviations: BMT, bone marrow transplant; CRP, C‐reactive protein; CT, computed tomography; EGD, esophagogastroduodenoscopy; GI, gastrointestinal; GVHD, graft‐vs‐host‐disease; HSCT, hematopoietic stem cell transplant; MRI, magnetic resonance imaging; PEG, percutaneous endoscopic gastrostomy; SDS, Shwachman–Diamond syndrome; WCE, Wireless capsule endoscopy.

^a^ Patients may have had more than one indication.

^b^ Mesenteric adenitis, dyspepsia, PEG replacement, pre‐screening for BMT.

^c^ Not mutually exclusive, patients could have more than one indication for endoscopy.

^d^ Immune suppression: calcineurin inhibitor (cyclosporine or tacrolimus), *mTor* inhibitor (sirolimus), *JAK* inhibitor (ruxolitinib).

^e^ Duodenal mass (*n* = 1), mesenteric adenitis (*n* = 1), fluid collection in right upper quadrant (*n* = 1). ^!^indicates the number of patients laboratory value was checked.

We identified 89 total indications for all endoscopic procedures (Table 2). These indications were not mutually exclusive. Diarrhea and/or steatorrhea were the most common indications for endoscopy derived from operative reports (*n* = 21 indications, 23.6%), followed by abdominal pain (*n* = 14 indications, 15.7%), FTT or weight loss (*n* = 14 indications, 15.7%) and nausea/vomiting (*n* = 10 indications, 11.2%). The majority of patients had endoscopic procedures performed for a single indication (*n* = 41 indications, 67.2%). In contrast, 20 patients (32.8%) had endoscopies performed for two or more indications.

**Figure 1:**
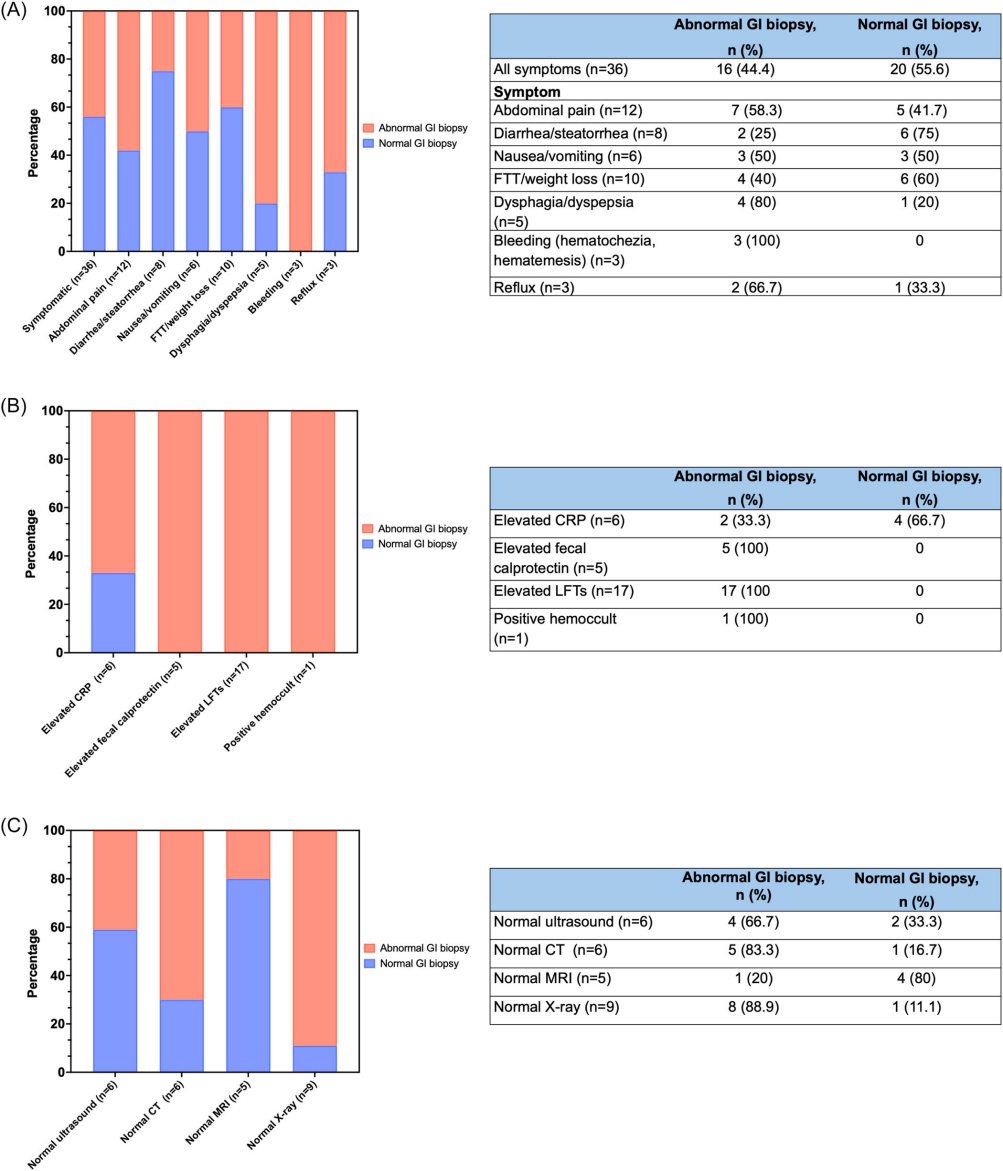
(A) Presenting gastrointestinal symptoms preceding endoscopy and corresponding abnormal (red) or normal (blue) biopsy findings are shown. Symptom data were available for 36 patients, in which abdominal pain was the most commonly reported symptom (*n* = 12, 33.3%), with abnormal biopsies in 58.3% (7/12). (B) The association between pre‐endoscopic laboratory abnormalities and GI biopsy findings are summarized. Abnormal GI biopsy findings were observed in 66.7% (2/3) of patients with elevated CRP (>2 mg/L), 100% (5/5) of patients with elevated fecal calprotectin, and 100% (17/17) of patients with elevated liver enzymes. (C) GI biopsy findings among patients with normal imaging performed within 3 months prior to endoscopy (*n* = 26/41, 63.4%), stratified by imaging modality are shown. Normal biopsy findings were observed in 33.3% (2/6) after ultrasound, 16.7% (1/6) following CT, 80.0% (4/5) following (MRI), and 11.1% (1/9) following X‐ray. Data are presented as number and percentage of patients. CRP, C‐reactive protein, CT, computed tomography, GI, gastrointestinal, MRI, magnetic resonance imaging.

We next examined the association between pre‐endoscopic symptoms, laboratory results, and imaging findings with gastrointestinal biopsy results. Endoscopic evaluation was performed in all patients based on clinical symptoms or indications; symptom data were available for 36 of the 45 patients who underwent endoscopy. In this group, 16 (44.4%) showed abnormal histopathologic findings on all endoscopic procedures (Figure 1A). Dysphagia/dyspepsia (*n* = 4, 80%) and gastrointestinal bleeding (*n* = 3, 100%) were associated with the highest rates of abnormal biopsy findings, whereas abdominal pain showed a more modest association (*n* = 7, 58.3%).

Elevated fecal calprotectin and liver enzymes were frequently associated with abnormal biopsy findings, suggesting that laboratory markers may help risk‐stratify patients undergoing endoscopy (Table 2). C‐reactive protein (CRP) was evaluated in 22 patients preceding endoscopy; among those with elevated CRP levels (CRP > 2 mg/L, *n* = 4 patients), abnormal endoscopic findings were observed in 66.7% of patients (Figure 1B). Fecal calprotectin was also not frequently obtained (*n* = 7 patients) but was associated with a high proportion of abnormal results when elevated (*n* = 5, 100%). Liver enzymes were the most commonly checked laboratory test (*n* = 54 patients) and when elevated, was associated with abnormal biopsy findings in all patients (*n* = 17, 100%). Median ALT level at the time of assessment was 166 U/L (5.5× ULN; range 37–516 U/L, 1.2–17.2× ULN). Imaging results were not reliably associated with the absence of gastrointestinal pathology on biopsy in our cohort, potentially suggesting that imaging should be interpreted with additional clinical and laboratory evaluation. Imaging was performed in 41 patients within 3 months preceding endoscopic procedures (Table 2). The majority of these images were normal (*n* = 26, 63.4%) or had evidence of pancreatic changes that are well‐documented in SDS (*n* = 10, 24.4%).^10^, ^11^ We evaluated whether normal imaging results could effectively exclude pathologic endoscopic findings (Figure 1C). Normal imaging was observed in six patients by ultrasound, six by CT, five by MRI, and nine by X‐ray. Among patients with normal ultrasound findings, two of six (33.3%) had no abnormalities on biopsy, compared with one of six (16.7%) following computed tomography (CT) and one of nine (11.1%) following X‐ray. In contrast, among patients who underwent MRI, four of five (80%) with normal imaging had normal biopsy findings.

**Figure 2:**
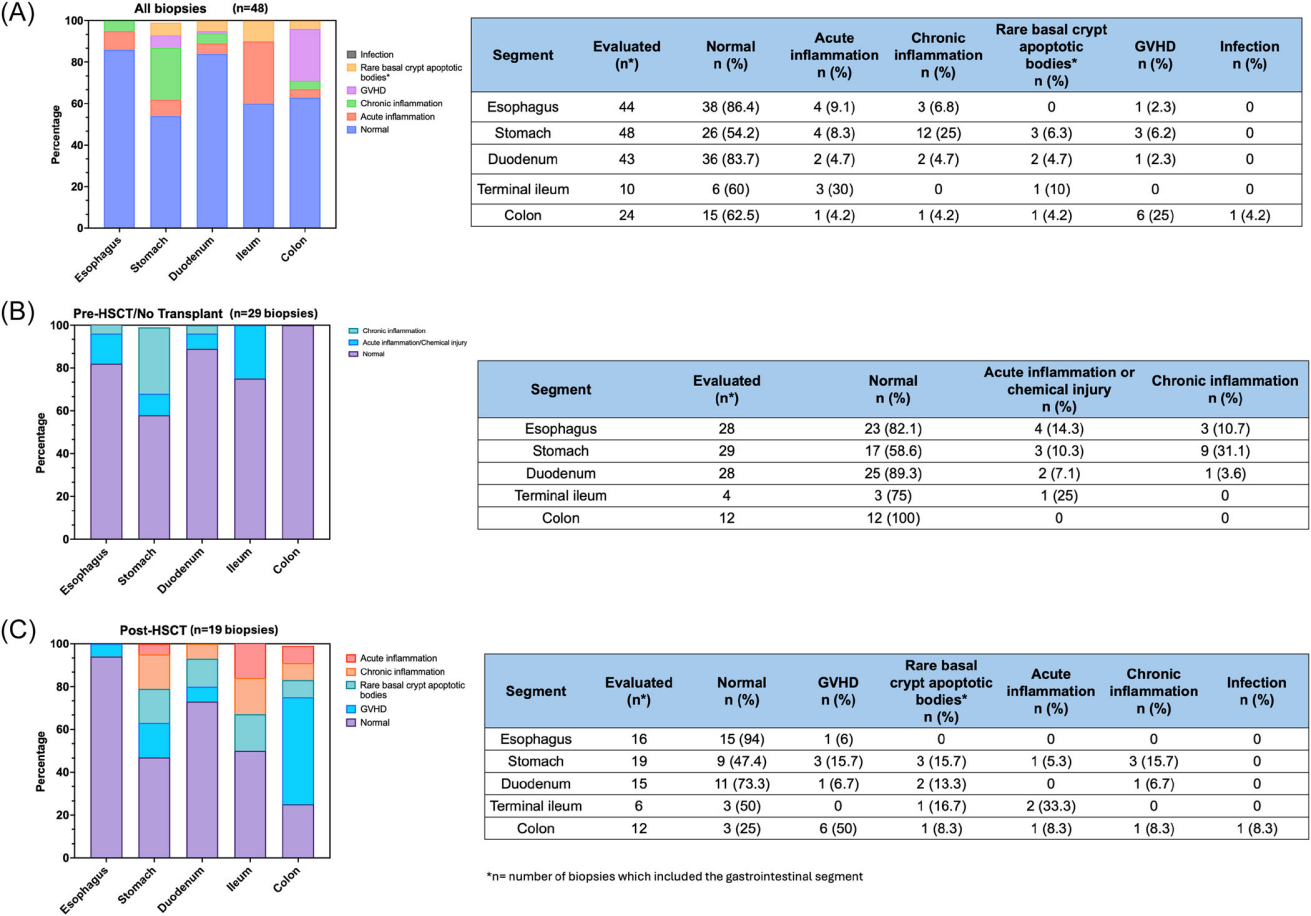
(A) The distribution of histopathologic findings across gastrointestinal segments for all available biopsies are described. Findings ranged from normal mucosa to GVHD associated changes. (B) Histopathologic findings from pre‐HSCT/untransplanted biopsies are shown. Pre‐HSCT/untransplanted biopsies demonstrated predominantly normal histology with limited findings of acute and chronic inflammatory changes observed across each gastrointestinal segment. (C) Histopathologic findings from post‐HSCT biopsies are demonstrated. Generally, post‐HSCT biopsies demonstrated a more heterogeneous distribution of abnormalities across gastrointestinal segments ranging from inflammatory changes to GVHD‐associated changes. Data are presented as number and percentage of biopsies. GVHD, graft‐vs‐host‐disease; HSCT hematopoietic stem cell transplant.

We next evaluated the histopathologic findings from biopsy reports stratified by gastrointestinal segment and transplantation status to determine to identify any patterns in patients with SDS (Figure 2). Normal histology predominated across all segments, regardless of timing relative to HSCT (Figure 2A). Among abnormal specimens, inflammatory changes—acute and chronic were more frequently observed, with variation by anatomic location.

We had 29 total pre‐HSCT biopsies available for analysis. Pre‐HSCT biopsies were largely normal, with limited acute and chronic inflammation involving the esophagus (acute 14.3%, chronic 10.7%), stomach (acute 10.3%, chronic 31.1%), and duodenum (acute 7.1%, chronic 3.6%), and minimal ileal (25%) or colonic involvement (Figure 2B). In contrast, post‐HSCT biopsies (*n* = 19 total available biopsies) demonstrated more frequent and heterogeneous abnormalities, including inflammation (stomach 21%, duodenum 6.7%, ileum 33.3%, colon 16.6%), basal crypt apoptosis (stomach 15.7%, duodenum 13.3%, ileum 16.7%, colon 8.3%), and GVHD‐associated changes—most prominent in the colon (50%) and stomach (15.7%)—with rare infectious findings (8.3%) (Figure 2C).

Centralized pathology review by a single blinded pathologist was performed for 19 cases from 13 unique patients with available pathology slides (Table S1). Across patients, most gastrointestinal biopsies were normal or were not available for analysis, with abnormalities primarily observed post‐transplant and involving inflammatory changes, GVHD, or infection. Notable findings included adenovirus infection in one patient (B‐2), mild to equivocal GVHD in several post‐transplant cases (E, F, K), and occasional gastritis, ileitis, colitis, or reactive changes, while pre‐transplant samples were largely normal. Representative images from biopsies are shown in Figure S1, demonstrating features consistent with GVHD in the colon and stomach (Figure S1A–C), adenoviral infection with associated inflammation in the ileum and colon (Figure S1D–F).

## 4 DISCUSSION

Herein, we characterize the largest cohort of genetically confirmed patients with SDS and summarize their endoscopic features. Our data suggest that routine endoscopy in asymptomatic or nonspecific symptomatic patients with SDS may have limited yield. In this cohort, endoscopic evaluation yielded a moderate overall diagnostic yield. However, the likelihood of abnormal findings varied by presenting symptom, preceding laboratory testing, and transplant status. In our cohort, preceding radiographic imaging was the least helpful in determining the potential diagnostic yield of endoscopy.

Our data augment the current understanding of endoscopic features within SDS. N Shah et al. previously reported inflammatory enteropathic changes in approximately 50% of duodenal biopsies from patients with SDS.^7^ In contrast, our multicenter registry study cohort demonstrated lower frequencies of duodenal inflammation, suggesting that clinically significant enteropathy may be less common than previously described.

In our cohort, certain symptoms (e.g., bleeding, dysphagia/dyspepsia, and abdominal pain) may represent higher‐risk phenotypes of gastrointestinal involvement in SDS that may warrant stronger consideration for endoscopy. Although limited by small sample size, these observations suggest that specific symptoms may identify patients with a higher likelihood of underlying gastrointestinal pathology. Furthermore, laboratory markers such as fecal calprotectin and liver enzymes when abnormal may help further risk‐stratify patients. However, the results from our cohort must be interpreted cautiously because of limited numbers. Our data suggest that routine endoscopic evaluation in the absence of concerning symptoms or supportive laboratory abnormalities may have limited diagnostic yield in patients with SDS.

We also observed differences in gastrointestinal pathology based on transplantation status. Endoscopic and histologic findings in post‐HSCT patients in our cohort were broadly consistent with those reported in other post‐HSCT pediatric populations, including mucosal erythema, villous atrophy, and histologic evidence of GVHD even in the absence of overt endoscopic abnormalities.^12^, ^13^ Notably, post‐HSCT biopsies also demonstrated a higher frequency of acute and chronic inflammatory changes and basal crypt apoptoses insufficient for a formal diagnosis of GVHD, a finding not observed in the pre‐HSCT/no‐transplant group. While these changes likely reflect transplant‐related processes such as conditioning toxicity, immunosuppression, and subclinical GVHD rather than SDS‐specific pathology, to our knowledge this represents the first description of the post‐HSCT GI histologic phenotype in patients with SDS and underscores the importance of distinguishing transplant‐related pathology from primary SDS‐associated gastrointestinal disease in this growing survivor population.

The endoscopic and histopathologic findings in our cohort have several potential clinical implications. In pre‐HSCT and non‐transplanted patients, the identification of esophageal, gastric, or duodenal inflammation may support the use of proton pump inhibitors or other targeted therapies. In post‐HSCT patients, the finding of basal crypt apoptosis or GVHD‐associated changes has direct implications for the adjustment of immunosuppression, while the identification of adenoviral infection on biopsy highlights the importance of routine viral staining and may prompt initiation or modification of antiviral therapy. Collectively, these findings underscore the clinical value of endoscopic evaluation with biopsy in symptomatic SDS patients, as histopathologic diagnosis can meaningfully guide management decisions that extend beyond the gastrointestinal tract.

Major limitations of this study are the availability and completeness of data inherent to registry‐based research. Another notable limitation is that histopathologic findings in the post‐HSCT subgroup (*n* = 19 biopsies) cannot be fully separated from transplant‐related effects, including GVHD, conditioning regimen toxicity, and immunosuppressive medications, all of which independently contribute to GI mucosal injury. As such, these findings should not be interpreted as reflecting SDS‐intrinsic GI pathology, and we addressed this by presenting pre‐HSCT/no‐transplant and post‐HSCT findings separately throughout. Nonetheless, these data represent the first description of the GI endoscopic and histologic phenotype in SDS patients following HSCT and may help generate hypotheses for future studies incorporating standardized GVHD grading and treatment data.

Other limitations include retrospective study design, small sample size and variable laboratory and radiographic work‐up conducted within this cohort. Furthermore, operative and pathology reports and clinical information about GVHD severity are not systematically collected in the registry and are dependent on available data and information provided by patients, families, and clinicians. Together, these limitations restrict our ability to provide definitive conclusions regarding predictive value for endoscopic evaluation in patients with SDS. A key strength of this study is that it represents, to our knowledge, the largest cohort of genetically confirmed SDS patients with systematically evaluated endoscopic findings.

## 5 CONCLUSION

In conclusion, endoscopic evaluation in patients with SDS yields abnormal histopathologic findings in a subset of cases, with diagnostic yield varying by indication, laboratory results and transplant status. Routine endoscopy in asymptomatic or nonspecifically symptomatic patients may have limited utility, whereas targeted evaluation based on symptom or supportive laboratory abnormalities may enhance diagnostic yield. These data provide contemporary insight into the gastrointestinal manifestations of SDS and may help inform clinical decision‐making regarding endoscopic population in this rare disease.

## CONFLICT OF INTEREST STATEMENT

The authors declare no conflicts of interest.

## Supporting information


**Supplementary Figure 1.** In the colon, mild graft‐versus‐host disease (GVHD) is characterized by preserved crypt architecture (A; H&E, 4×) with increased basal crypt apoptoses (B; H&E, 10×). In the stomach, GVHD changes are more subtle, manifested by rare mid‐zone glandular apoptotic bodies (arrow) (C; H&E, 20×). In one patient, adenovirus infection involving the ileum and colon is identified, characterized by viral cytopathic changes in the surface epithelium (D; H&E, 10×), confirmation by adenovirus immunohistochemistry (E; 10×), and associated acute inflammation/cryptitis in adjacent mucosa (F; H&E, 10×).

Supporting file 1.

## Data Availability

The data that support the findings of this study are available on request from the corresponding author. The data are not publicly available due to privacy or ethical restrictions.

## References

[1] Nelson A , Myers K . Shwachman‐diamond syndrome. In: Adam MP , Feldman J , Mirzaa GM , Pagon RA , Wallace SE , Amemiya A , eds. GeneReviews® [Internet]. University of Washington, Seattle; 1993. 20301722

[2] Nelson AS , Myers KC . Diagnosis, treatment, and molecular pathology of Shwachman‐Diamond syndrome. Hematol Oncol Clin North Am. 2018;32(4):687‐700. 30047420 10.1016/j.hoc.2018.04.006

[3] Boocock GRB , Morrison JA , Popovic M , et al. Mutations in SBDS are associated with Shwachman‐Diamond syndrome. Nature Genet. 2003;33(1):97‐101. 12496757 10.1038/ng1062

[4] Myers K , Hebert K , Antin J , et al. Hematopoietic stem cell transplantation for Shwachman‐Diamond syndrome. Biol Blood Marrow Transplant. 2020;26(8):1446‐1451. 32428734 10.1016/j.bbmt.2020.04.029 PMC7371524

[5] Veropalumbo C , Campanozzi A , De Gregorio F , Correra A , Raia V , Vajro P . Shwachman‐Diamond syndrome with autoimmune‐like liver disease and enteropathy mimicking celiac disease. Clin Res Hepatol Gastroenterol. 2015;39(1):e1‐e4. 25129842 10.1016/j.clinre.2014.06.017

[6] Nissen LHC , Stuurman KE , van der Feen C , Kemperman FA , Pruijt JFM , de Jonge HJM . Inflammatory bowel disease in Shwachman‐Diamond syndrome; is there an association? Clin Res Hepatol Gastroenterol. 2020;44(1):e10‐e13. 31196706 10.1016/j.clinre.2019.05.006

[7] Shah N , Cambrook H , Koglmeier J , et al. Enteropathic histopathological features may be associated with Shwachman‐Diamond syndrome. J Clin Pathol. 2010;63(7):592‐594. 20501449 10.1136/jcp.2010.077677

[8] Schwimmer JB , Dunn W , Norman GJ , et al. SAFETY study: alanine aminotransferase cutoff values are set too high for reliable detection of pediatric chronic liver disease. Gastroenterology. 2010;138(4):1357‐1364.e2. 20064512 10.1053/j.gastro.2009.12.052 PMC2846968

[9] Dong MH , Bettencourt R , Barrett‐Connor E , Loomba R . Alanine aminotransferase decreases with age: the Rancho Bernardo Study. PLoS One. 2010;5(12):e14254. 21170382 10.1371/journal.pone.0014254 PMC2999530

[10] Adachi M , Tachibana K , Asakura Y , Aida N . Usefulness of pancreatic ultrasonography in the diagnosis of Shwachman‐Bodian‐Diamond syndrome. Acta Paediatr (Stockholm). 2005;94(11):1686‐1690. 10.1080/08035250510034932 16303713

[11] Berrocal T , Simón MJ , al‐Assir I , et al. Shwachman‐Diamond syndrome: clinical, radiological and sonographic findings. Pediatr Radiol. 1995;25(5):356‐359. 7567263 10.1007/BF02021702

[12] Slae M , Ben‐Or MS , Schwell R , et al. Endoscopic findings in the evaluation of gastrointestinal symptoms in pediatric patients post hematopoietic stem cell transplantation. J Pediatr Gastroenterol Nutr. 2025;81(1):131‐139. 40313112 10.1002/jpn3.70051

[13] Slae M , Pinhasov D , Averbuch D , et al. Evaluation of gastrointestinal symptoms in pediatric patients post hematopoietic stem cell transplantation: ileo‐colonoscopy versus sigmoidoscopy. A single‐center experience and review of literature. Pediatr Blood Cancer. 2021;68(11):e29235. 34264544 10.1002/pbc.29235

