# Supplementary material for: Endoscopic findings in patients with Shwachman–Diamond syndrome: A report from the North American Shwachman–Diamond syndrome registry
Source: JPGN Rep. 2026 Jul 5;7(4):601–8. doi: 10.1002/jpr3.70218 (PMC13399004; doi:10.1002/jpr3.70218)

Supplementary Figure 1. Spectrum of gastrointestinal pathology in Shwachman-Diamond Syndrome

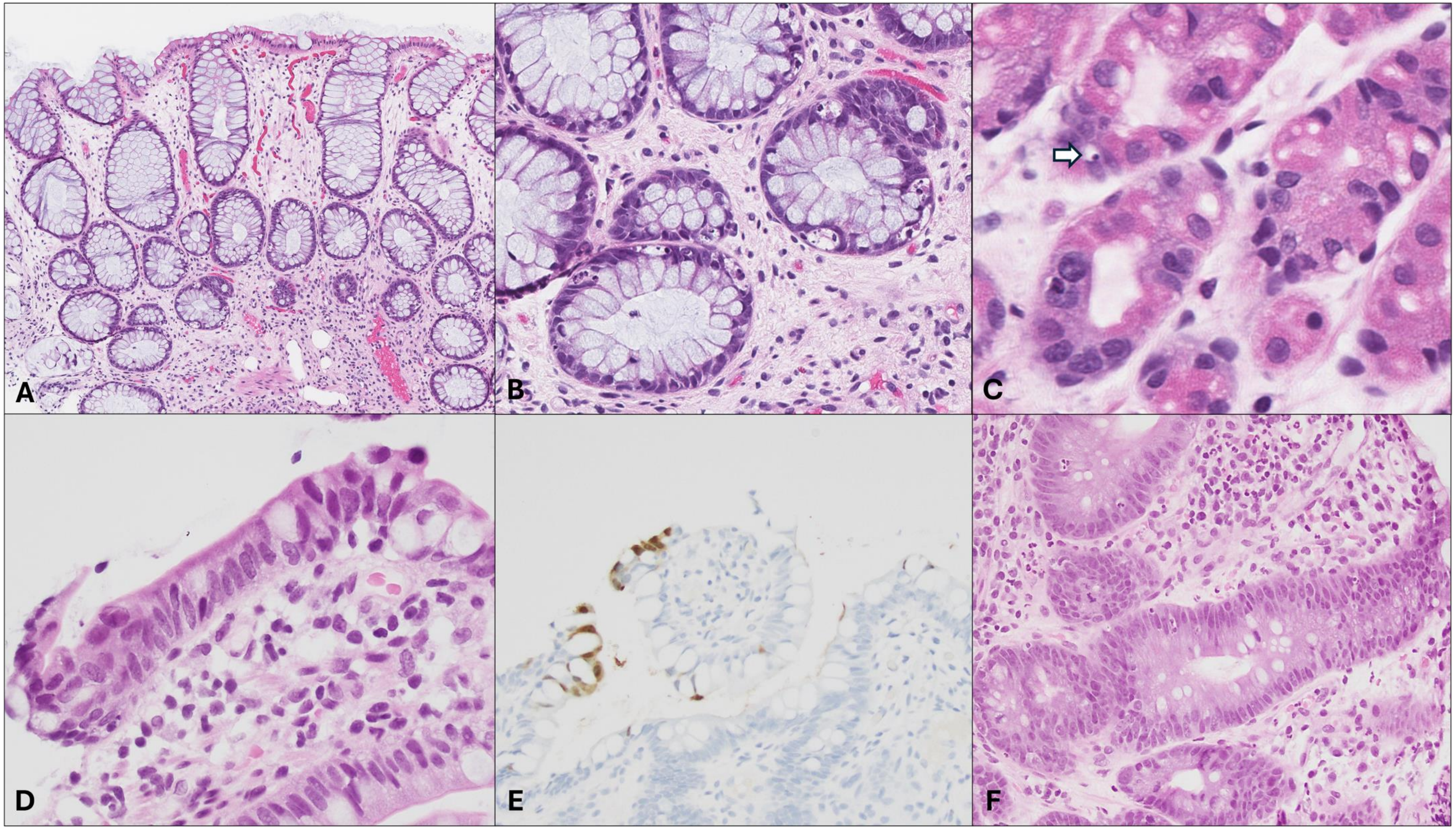

Supplement: Supplementary file 1 [file JPR3-7--s001.pdf]
