# Supplementary material for: Endoscopic findings in patients with Shwachman–Diamond syndrome: A report from the North American Shwachman–Diamond syndrome registry
Source: JPGN Rep. 2026 Jul 5:10.1002/jpr3.70218. Online ahead of print. doi: 10.1002/jpr3.70218 (PMC13399004; doi:10.1002/jpr3.70218)

Supplementary Figure 1. Spectrum of gastrointestinal pathology in Shwachman-Diamond Syndrome

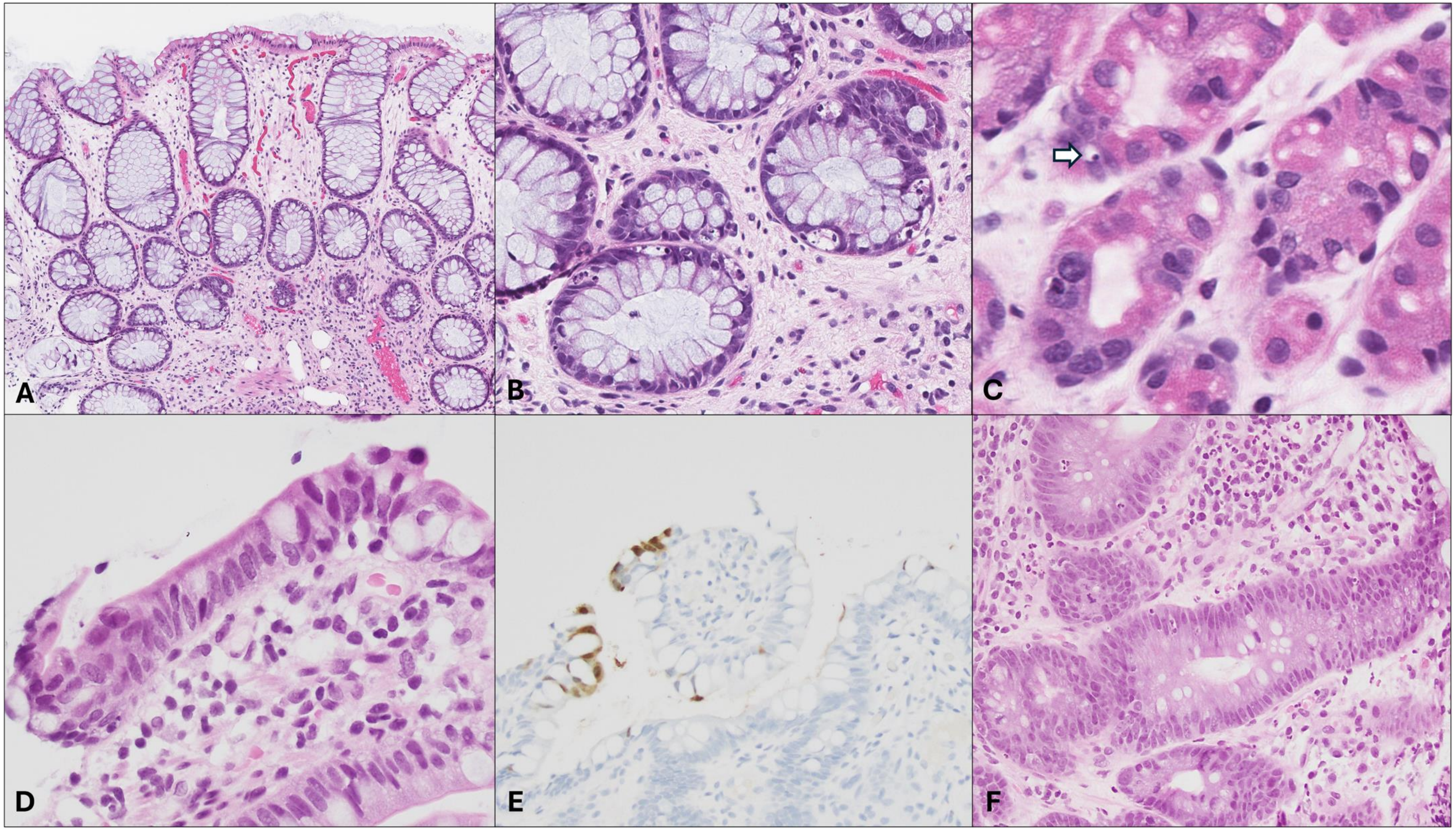

Supplement: Supplementary file 1 — Supplementary Figure 1. In the colon, mild graft‐versus‐host disease (GVHD) is characterized by preserved crypt architecture (A; H&E, 4×) with increased basal crypt apoptoses (B; H&E, 10×). In the stomach, GVHD changes are more subtle, manifested by rare mid‐zone glandular apoptotic bodies (arrow) (C; H&E, 20×). In one patient, adenovirus infection involving the ileum and colon is identified, characterized by viral cytopathic changes in the surface epithelium (D; H&E, 10×), confirmation by adenovirus immunohistochemistry (E; 10×), and associated acute inflammation/cryptitis in adjacent mucosa (F; H&E, 10×). [file JPR3-9999-0-s002.pdf]
