# Supplementary material for: Endoscopic findings in patients with Shwachman–Diamond syndrome: A report from the North American Shwachman–Diamond syndrome registry
Source: JPGN Rep. 2026 Jul 5:10.1002/jpr3.70218. Online ahead of print. doi: 10.1002/jpr3.70218 (PMC13399004; doi:10.1002/jpr3.70218)
Supplement: Supplementary file 2 — Supporting file 1. [file JPR3-9999-0-s001.docx]

**Supplementary Table 1. Summary of gastrointestinal pathology findings by central review**

|  |  | **Esophagus** | | | **Stomach** | | **Duodenum** | **Ileum** | **Cecum** | **Ascending colon** | **Transverse colon** | **Descending colon** | **Sigmoid** | **Rectum** |
| --- | --- | --- | --- | --- | --- | --- | --- | --- | --- | --- | --- | --- | --- | --- |
| **Patient ID** | **Time from HSCT** | **Distal** | **Mid** | **Proximal** | **Corpus** | **Antrum** |  |  |  |  |  |  |  |  |
| **A** | Pre-transplant | NA | NA | NA | NA | Normal | Normal | NA | NA | NA | NA | NA | NA | NA |
| **B-1** | NA | Normal | NA | NA | Normal | NA | Normal | NA | NA | NA | NA | NA | NA | NA |
| **B-2** | NA | Normal | Normal | NA | Normal | Normal | Normal | Erosion, increased basal crypt apoptoses and adenovirus infection | Positive adenovirus IHC | Normal | Positive adenovirus IHC | Positive adenovirus IHC | Positive adenovirus IHC | Positive adenovirus IHC |
| **C-1** | NA | Normal | NA | NA | Normal | Normal | Normal | Normal | NA | Normal | NA | Normal | NA | Normal |
| **C-2** | NA | Normal | NA | Normal | Normal | Normal | Normal | NA | NA | NA | NA | NA | Normal | Focal acute inflammation; negative adenovirus IHC |
| **D** | NA | Normal | Normal | NA | Normal | Normal | Normal | Abundant lamina propria macrophages | Normal | NA | Normal | NA | Normal | Normal |
| **E-1** | Post-transplant | Normal | NA | NA | Normal | Normal | NA | NA | NA | NA | NA | NA | NA | Mild GVHD |
| **E-2** | Post-transplant | Normal | NA | NA | Equivocal for GVHD | Normal | NA | NA | NA | NA | NA | NA | NA | Mild GVHD |
| **E-3** | Post-transplant | Normal | NA | NA | Normal | Normal | NA | NA | NA | NA | NA | NA | NA | Normal |
| **F-1** | Post-transplant | Normal | NA | NA | NA | Normal | Normal | Normal | NA | Mild GVHD | NA | Equivocal for GVHD | NA | Normal |
| **F-2** | Post-transplant | Normal | NA | Normal | Normal | Normal | Normal | Normal | NA | Normal | NA | Normal | NA | Normal |
| **G** | Post-transplant | Normal | NA | Normal | Normal | Normal | Normal | NA | NA | NA | NA | NA | NA | NA |
| **H** | Pre-transplant | Normal | NA | NA | Reactive gastropathy | NA | NA | NA | NA | NA | NA | NA | NA | Normal |
| **I-1** | Post-transplant | Normal | NA | Normal | Normal | Normal | NA | NA | NA | NA | NA | NA | NA | Normal |
| **I-2** | Post-transplant | Normal | NA | Normal | Chronic focally active gastritis | NA | NA | Moderately active ileitis with a non-necrotizing granuloma | NA | Normal | NA | Normal | NA | Normal |
| **I-3** | Post-transplant | NA | NA | NA | Chronic inactive gastritis | Chronic focally active gastritis | Normal | NA | NA | NA | NA | NA | NA | NA |
| **J** | Post-transplant | Normal | NA | NA | Normal | NA | NA | NA | NA | NA | NA | NA | NA | Focal mildly active colitis; No GVHD |
| **K** | Post-transplant | Normal | NA | Normal | Normal | Normal | Normal | Mild active ileitis | NA | Normal | NA | Mild GVHD | NA | Mild GVHD |
| **L** | Post-transplant | Normal | NA | Normal | Normal | Normal | Normal | NA | NA | NA | NA | NA | NA | NA |

HSCT: hematopoietic stem cell transplant; NA: not applicable; IHC: immunohistochemistry; GVHD: graft-vs-host disease
